# Gene Expression Ratios Lead to Accurate and Translatable Predictors of DR5 Agonism across Multiple Tumor Lineages

**DOI:** 10.1371/journal.pone.0138486

**Published:** 2015-09-17

**Authors:** Anupama Reddy, Joseph D. Growney, Nick S. Wilson, Caroline M. Emery, Jennifer A. Johnson, Rebecca Ward, Kelli A. Monaco, Joshua Korn, John E. Monahan, Mark D. Stump, Felipa A. Mapa, Christopher J. Wilson, Janine Steiger, Jebediah Ledell, Richard J. Rickles, Vic E. Myer, Seth A. Ettenberg, Robert Schlegel, William R. Sellers, Heather A. Huet, Joseph Lehár

**Affiliations:** 1 Novartis Institutes for Biomedical Research, Cambridge, MA, United States of America; 2 Horizon CombinatoRx, Cambridge, MA, United States of America; University of Nebraska Medical Center, UNITED STATES

## Abstract

Death Receptor 5 (DR5) agonists demonstrate anti-tumor activity in preclinical models but have yet to demonstrate robust clinical responses. A key limitation may be the lack of patient selection strategies to identify those most likely to respond to treatment. To overcome this limitation, we screened a DR5 agonist Nanobody across >600 cell lines representing 21 tumor lineages and assessed molecular features associated with response. High expression of DR5 and Casp8 were significantly associated with sensitivity, but their expression thresholds were difficult to translate due to low dynamic ranges. To address the translational challenge of establishing thresholds of gene expression, we developed a classifier based on ratios of genes that predicted response across lineages. The ratio classifier outperformed the DR5+Casp8 classifier, as well as standard approaches for feature selection and classification using genes, instead of ratios. This classifier was independently validated using 11 primary patient-derived pancreatic xenograft models showing perfect predictions as well as a striking linearity between prediction probability and anti-tumor response. A network analysis of the genes in the ratio classifier captured important biological relationships mediating drug response, specifically identifying key positive and negative regulators of DR5 mediated apoptosis, including DR5, CASP8, BID, cFLIP, XIAP and PEA15. Importantly, the ratio classifier shows translatability across gene expression platforms (from Affymetrix microarrays to RNA-seq) and across model systems *(in vitro* to *in vivo*). Our approach of using gene expression ratios presents a robust and novel method for constructing translatable biomarkers of compound response, which can also probe the underlying biology of treatment response.

## Introduction

Death Receptor 5 (DR5, TNFRSF10B), a receptor for Apo2L ligand or Tumor Necrosis Factor (TNF)–Related Apoptosis Inducing Ligand (Apo2L/TRAIL), signals through apoptotic pathways to induce cell death [1]. Multiple therapeutic agonists of DR5, including antibodies and recombinant Apo2L/TRAIL, have been developed and evaluated clinically in unselected patient populations [2]. Despite significant anti-tumor activity in preclinical models, efficacy in clinical settings has been disappointing. While these agents have been generally well tolerated, durable responses to monotherapy have been reported in only a few patients [3,4]. Thus, new approaches to targeting and predicting response to DR5 activation are needed to improve upon current therapeutics.

Activation of DR5 leads to recruitment of the adaptor protein Fas-associated death domain (FADD) to the intracellular death domain of DR5 to form the death inducing signaling complex (DISC) [1]. Once bound in the DISC, initiator caspase-8 is cleaved, resulting in activation of downstream effector caspases (i.e., caspase-3 and -7) to drive the extrinsic apoptotic program. In so-called type II cells, caspase-8 cleaves BID to induce mitochondrial-dependent, intrinsic apoptotic signaling [5]. Several inhibitory proteins, such as c-FLIP, which negatively regulates caspase-8, and the IAP and Bcl-2 protein families keep the apoptotic program in check [1]. The heterogeneous expression of these and other pro-survival signaling factors suggests that multiple molecular features might contribute to mediating the response to DR5-mediated apoptosis [6], highlighting the complexity of predicting response.

To date, DR5 targeting efforts have focused primarily on bivalent antibodies which depend on secondary Fc-mediated crosslinking for activity by immune cells [7–9]. We previously reported a novel, more potent tetravalent DR5 agonist Nanobody, DR5Nb1-tetra [10]. Nanobodies are a class of therapeutic proteins derived from the variable domains (V_HH_) of heavy chain-only antibodies that occur naturally in camelidae family [11]. In addition to increased valency, DR5Nb1-tetra induces apoptosis independent of exogenous cross-linking, and thus, like small molecule compounds, is amenable to high-throughput screening. Using DR5Nb1-tetra, we investigated a novel gene ratio expression classifier built using response data from 600 cell lines, and validated it in an independent set of patient-derived tumor xenograft (PTX) models. These data suggest that a gene-expression ratio-based classifier that incorporates the biological relationships between pathway genes is a robust method for predicting drug response, and importantly, has potential for clinical translation.

## Results

### Pan-cancer in vitro screening of the DR5 agonist

**Fig 1:**
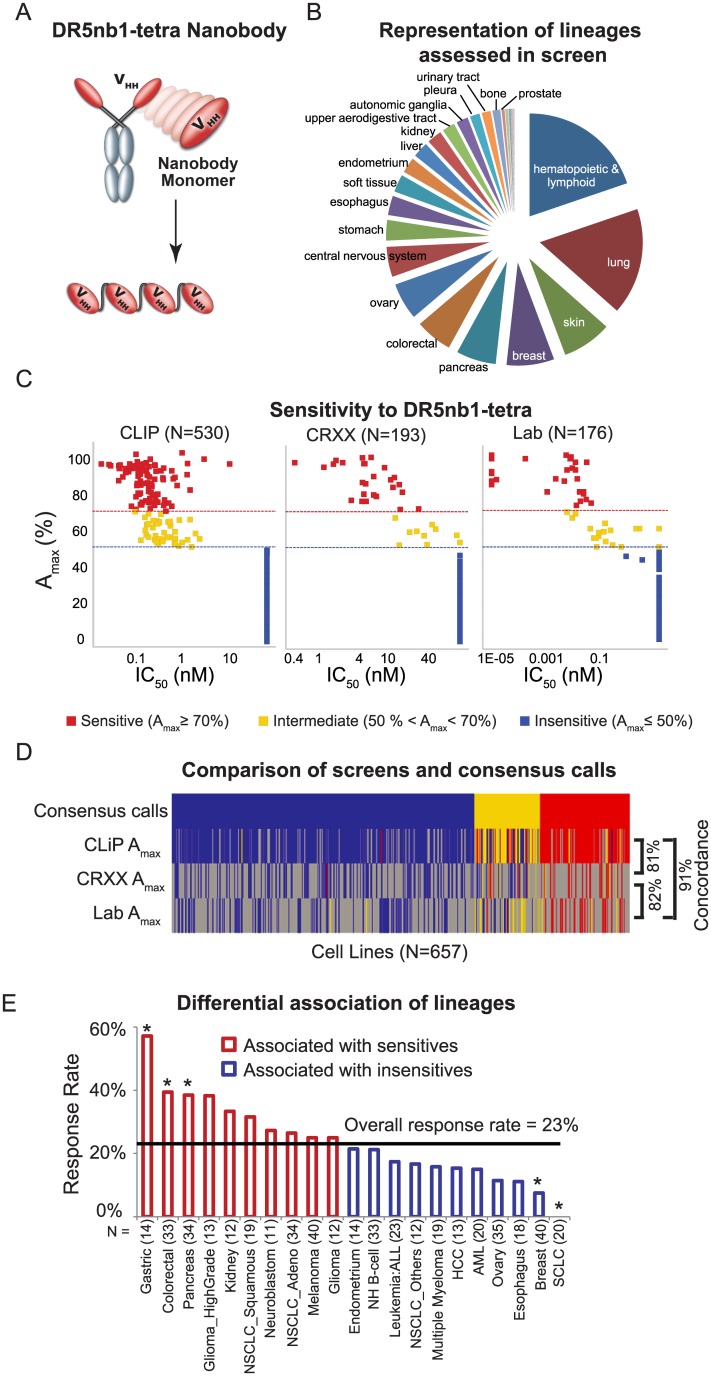
DR5Nb1-tetra is selective with responses in multiple tumor lineages. (*A*) Schematic diagram of DR5Nb1-tetra Nanobody. *(B)* Composition of *in vitro* pan-cancer screen tested for response to DR5Nb1-tetra. *(C)* DR5Nb1-tetra response in the CLiP, CRXX and Lab screens. Response is shown as A_max_ relative to IC_50_. A_max_ cut-offs for sensitive, intermediate, and insensitive classes are drawn. (*D*) Consistency of A_max_ values across the three screens. A_max_ values for each screen (CLIP, CRXX and Lab) are shown as a heatmap colored to represent sensitive (red), intermediate (yellow) and insensitive (blue) categories defined using the same thresholds for A_max_ across the three screens. Missing values are shown in gray. (*E*) Response rates (% sensitives) are plotted for each of the lineages (#cell lines ≥10). Lineages with significant (p<0.05 using Fisher’s exact test) enrichment are denoted by *.

DR5Nb1-tetra is a potent Nanobody agonist of DR5 [10] (Fig 1A). The increased potency of DR5Nb1-tetra and lack of dependency on secondary crosslinking enabled us for the first time to assess the sensitivity to DR5 agonists across a broad spectrum of cancer lineages in high throughput format. DR5Nb1-tetra sensitivity was evaluated in three independent, large-scale *in vitro* screens, designated CLiP, CRXX, and Lab, comprised of 530, 193 and 178 cell lines, respectively, to measure cell viability. The CLiP and CRXX screens were automated in 1,536-well and 384-well plates, respectively. The Lab screen was manually executed in 96-well plates. The final set included 80% solid tumor cell lines, with over 25 tumor lineages represented (Fig 1B, S1 Fig, S1 File). Responses to DR5Nb1-tetra were characterized using sigmoidal fits to each cell line’s dose response values, and recording the maximum % inhibition (A_max_) and the dose required for 50% growth inhibition (IC_50_) measurements (S2 Fig). For cell lines which had a maximum inhibition <50%, we assign an IC_50_ corresponding to the maximum dose. Within each screen, we observed a strong separation of response, with a >10x dynamic range between sensitives and insensitives (Fig 1C).

Despite slight differences in the formats of the high-throughput assays used in these screens (plate size, concentration ranges, etc.), the screening results were found to be highly concordant. Sensitivity cutoffs were defined within each screen based on A_max_ value, resulting in A_max_ ≥70% for sensitive; A_max_ 50% to 70% intermediate; and A_max_ ≤50% insensitive. As sensitivity calls were highly concordant (>80%) in pair-wise comparison of cell lines common to two screens, the data from all screens could be aggregated, resulting in higher confidence sensitivity calls (Fig 1D). Consensus sensitivity calls were then calculated by majority voting across the three screens, leading to a total of 657 distinct cell lines, with a 23% sensitivity rate. After removing cell lines with conflicting and intermediate calls, the aggregated data (N = 558) covered 21 tumor lineages with at least 10 cell lines per lineage. Lineages were ranked based on percentage of sensitives, and analyzed for significance of enrichment of sensitivity using Fisher’s exact test (Fig 1E). The top three most responsive lineages, gastric, colorectal and pancreatic cancer, were significantly associated with sensitivity (P<0.05). The two least responsive lineages, breast and small cell lung cancer, were significantly associated with insensitivity (P<0.05).

### Correlation analysis for identifying top ranking features associated with sensitivity

**Fig 2:**
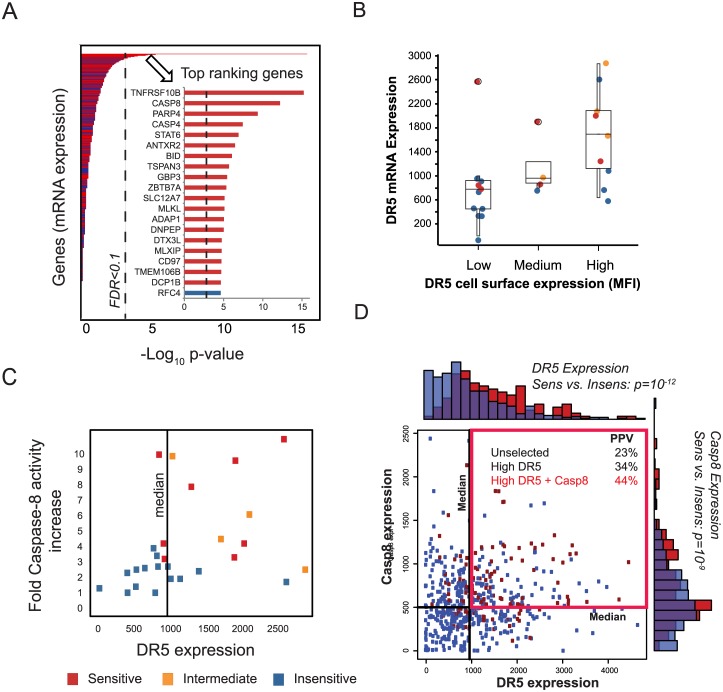
DR5 and CASP8 expression are top ranking features correlated with sensitivity and together improve predictions. (*A*) Differential association of gene expression using p-values to assess the significance of gene expression features correlated with DR5Nb1-tetra sensitivity. Inset shows the top 20 genes (dotted line = FDR < 0.05). Colors are based on association with sensitives (red) and insensitives (blue). (*B*) Comparison of relative surface protein levels of DR5 to mRNA expression in 25 pancreatic cancer cell lines. Points are colored based on sensitivity to DR5Nb1-tetra: sensitives (red), intermediates (orange) and insensitives (blue). *(C)* Induction of Casp8 activity compared to DR5 gene expression in 27 pancreatic cell lines. (*D*) DR5+Casp8 expression compared to sensitivity. DR5 and Casp8 are individually significantly associated with response, as shown in the marginal histograms for sensitives (red), insensitives (blue), with overlap shown in purple (DR5: p = 10^−12^, Casp8: p = 10^−9^). Scatterplot of DR5 and Casp8 shows that cell lines with high expression of both genes (marked by red box) are enriched in sensitives (PPV = 44%) compared to high DR5 (PPV = 34%) and unselected (PPV = 34%).

Many factors could affect DR5Nb1-tetra sensitivity, so we explored associations with genetic alterations using data from the Cancer Cell Line Encyclopedia (CCLE) [12]. Notably, differential analysis of copy number (CN) features showed that high CN of the 8p21.3 chromosomal region (containing DR5) was significantly associated with sensitivity (S3A Fig). Several mutated genes, CDKN2A, MET, KRAS, TP53, were nominally significant (p<0.05), but did not pass the FDR<0.1 multiple hypothesis test (S3B Fig). For CCLE microarray gene expression features, the first and second ranked genes significantly associated with response (Student’s t-test) were TNFRSF10B (DR5) and CASP8, respectively (Fig 2A, S1 File). Pathway analysis of significant genes associated with response (FDR<0.1) revealed enrichment of the apoptosis pathway (S1 Table). Notably, we did not observe significant differential expression of other genes (GALNT14, STK17B, SP140L, AIM1 and SIX1) previously reported to be associated with sensitivity to DR5 agonists [13–15].

DR5 protein levels correlated well with mRNA expression when tested using 25 pancreatic cancer cell lines (Fig 2B). Cell surface expression was measured by flow cytometry and represented as mean fluorescent intensity (MFI) relative to COLO205. COLO205 was selected for normalization because it had medium mRNA expression of DR5 and CASP8. Cell lines with MFI within 30% of Colo205 were considered medium protein level. Greater than 30% were considered high, and less than 30% were considered low protein levels.

DR5 expression is a significant DR5Nb1-tetra response predictor that is necessary, but not sufficient for sensitivity to cell killing. We hypothesize that a minimum threshold of DR5 expression is required for pathway activation. In order to test this hypothesis, we tested caspase-8 activity induction by DR5Nb1-tetra in 27 pancreatic cancer cell lines with differential DR5 expression (Fig 2C). In general, responsive lines demonstrated increases in Caspase-8 activity, but activity was not sufficient for response in all models. Furthermore, most responsive lines, including intermediate responders, exceeded the median DR5 expression of the set. However, caspase-8 activity induction showed no dependence on DR5 expression among the sensitive lines, consistent with a threshold of DR5 expression being required. Expression of CASP8 was the second ranked feature correlated with response. When combined with DR5 in a 2-gene classifier, in which expression of DR5 and CASP8 above the median expression values predicts sensitivity, CASP8 improves response prediction accuracy (Fig 2C, S1 File). The performance of this predictor can be evaluated by computing the positive predictive value (PPV = (number of predicted sensitives)/(number of sensitives)) as an indicator of the expected response rate. This 2-gene classifier enriches for sensitives (PPV = 44%, AUC = 66%) compared to high DR5 alone (PPV = 34%, AUC = 64%) or no selection (PPV = 23%) (Fig 2C).

As DR5 expression has not typically been reported as a strong predictor of DR5 agonist sensitivity [6], we investigated whether this discrepancy is related to sample size in screening sets. The prediction significance was computed for all genes in sets of 100 or 200 randomly selected cell lines. Both DR5 and CASP8 show an average rank of ~1000 (S4 Fig), with DR5 nominally significant (P<0.05) in only ~25% and CASP8 in only ~10% of resampling simulations. Moreover, both genes have low dynamic ranges of expression (<2 fold), making it challenging to identify and translate thresholds for response predictive expression features (Fig 2C). Together, these findings suggest that prior studies failed to identify DR5 and Caspase-8 as response biomarkers because of their limited dynamic range compounded by small sample sizes.

### Gene expression ratio predictor for classifying DR5Nb1-tetra responses

**Fig 3:**
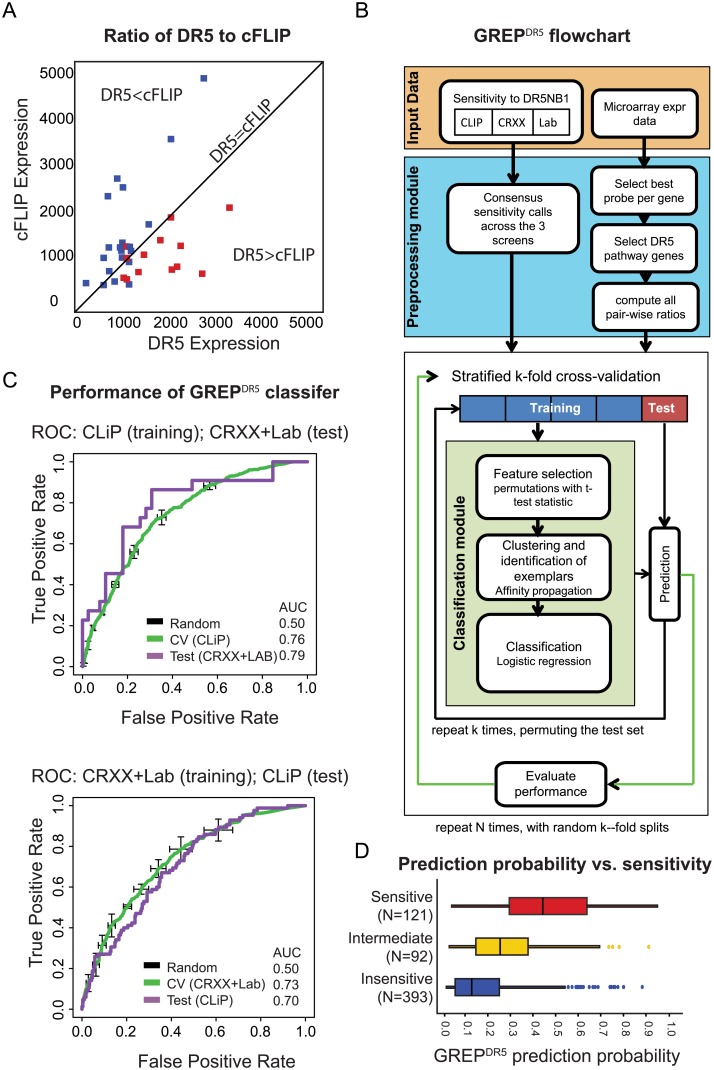
Gene expression ratio predictor (GREP). (*A*) Comparison of DR5 versus cFLIP expression in sensitive (red) and insensitive (blue) pancreatic cell lines shows their opposing effects on response. DR5>cFLIP explains sensitivity better than high DR5 alone. (*B*) Flowchart of analysis steps used for our GREP^DR5^ analysis. CLIP, CRXX and Lab indicate independent *in vitro* screens described in text. *(C)* Comparison of GREP performance in cross-validation (CV) between cell lines from a screen (training set) and tested in a completely independent screen (Test), using the CLiP and combined CRXX+Lab screens. Each chart shows the reciever-operator curves for prediction fidelity, for CV and Test runs, first using CLiP and then using CRXX+Lab as the training set. To preserve complete independence for the “Test” analyses, all lines common to the two screen sets were removed from the test set. (*D*) GREP^DR5^ prediction probability across sensitive (red), intermediate (yellow) and insensitive (blue) cell lines defined by a prediction threshold of 0.5.

While significantly enriching for sensitive cell lines, the DR5+CASP8 signature poses challenges for clinical translation. Ratios, which measure relative signals, can normalize sample-to-sample variation, reduce systematic noise, reduce biases introduced by model systems, and may better reflect the underlying biology of signaling pathways that are governed by relative levels of activated proteins [16]. Consistent with the known negative regulation of DR5 by cFLIP (CFLAR) [6], the DR5/cFLIP expression ratio is a relevant exemplar in our data (Fig 3A). While DR5/cFLIP discriminates sensitivity well in pancreatic cell lines, it fails as a predictor in other lineages. Thus, we sought to determine if accuracy in the pan-cancer setting may be improved by using multiple ratios.

We have developed a method termed Gene Ratio Expression Prediction (GREP), to generate classifiers built from gene expression ratios, and applied it to predict responses to DR5Nb1-tetra. The GREP method includes: calculating all pairwise expression ratios for a chosen set of genes across all samples; selecting representative ratios from similarity clustering; using logistic regression to build a predictor under cross-validation; and applying that model to predict sensitivity for each sample based on its observed gene expression ratios (Fig 3B). To limit computational costs and multiple hypothesis false discovery rates (expression array experiments have >10^8^ ratios), we focused on 173 genes with reported relevance to DR5 signaling or sensitivity to a DR5 agonist, or involvement in death domain caspase signaling (S2 Table). Correlations with sensitivity among the ~2,500 possible ratios were deemed significant if they exceeded (FDR<0.1) those found in simulations that used the same data with sample labels randomly permuted while maintaining mutual gene expression correlations. Affinity propagation clustering [17] was used to identify highly correlated clusters of these significant gene ratios, and representatives from each cluster were used to classify the samples using logistic regression (S1 File, S7 Fig). GREP^DR5^ prediction calls for all CCLE lines are reported in S1 File.

To evaluate the performance of GREP^DR5^ we have used cross-validation and independent test sets. Cross-validation is a technique for validating a predictive model to assess how well the results will perform on an independent dataset. In cross-validation studies, GREP^DR5^ performed well across lineages, and within specific lineages, prediction performance did not depend on the response rate (S5 Fig). The performance of GREP^DR5^ was also confirmed in an independent test set by partitioning the cell line responses into two sets: CLIP, CRXX+Lab, and using one of the sets as training and the non-overlapping cell lines from the other set as an independent test set (Fig 3C). GREP^DR5^ had significant performance in the independent test sets, and as expected, the performance on the test sets was similar to cross-validation performance on the training sets. Cross-validation ROC curves for CRXX+Lab have a higher variability compared to CLIP, because the sample set for CRXX+Lab (N = 158) is much smaller than CLIP (N = 434). Finally, GREP^DR5^ (PPV = 55%, AUC = 78%) outperforms random predictions (PPV = 23%, AUC = 57%) and the 2-gene signature (PPV = 44%, AUC = 66%). Important for translatability, most of the insensitive cell lines have lower prediction probabilities (median = 0.1), than sensitive cell lines (median = 0.45) representing a significant improvement (Student’s t-test p-value = 2x10^-24^) (Fig 3D).

### Translation of GREP^DR5^ predictions across model systems and across platforms

We sought to validate the performance of the GREP^DR5^ predictor and assess its translatability to an *in vivo* system. The anti-tumor activity of DR5Nb1-tetra was previously tested in 11 pancreatic PTX models [10]. Briefly, to maximally differentiate both efficacy and response, cohorts of mice were treated with high dose DR5Nb1-tetra (40 mg/kg weekly or 20 mg/kg bi-weekly) and tumor stasis (%T/C <10%) or regression (Regression <10%) relative to the vehicle control was used to classify tumor models as responders (S6 Fig). The overall response rate to DR5Nb1-tetra was 37%, similar to the response rate in pancreatic cell lines (Fig 1E).

**Fig 4:**
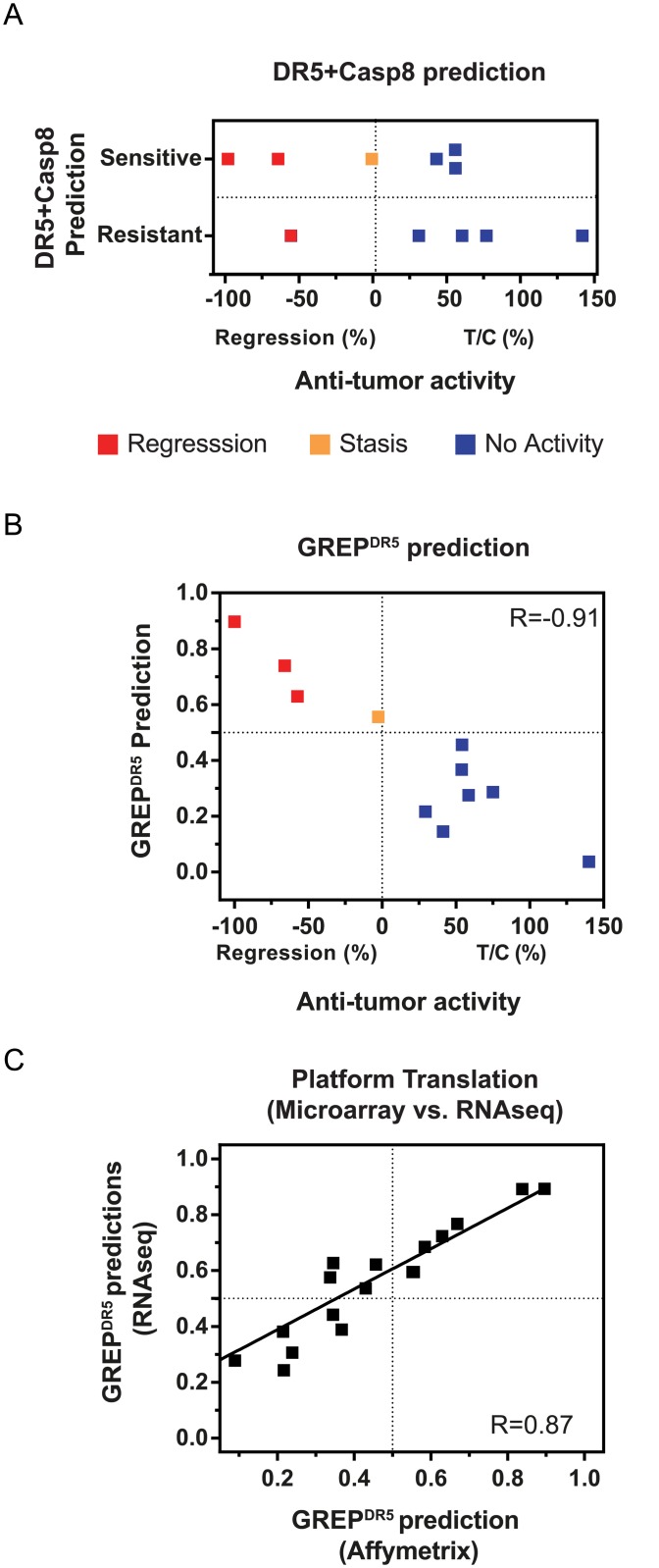
GREP^DR5^ accurately translates across model systems (*in vitro* to *in vivo*) and platforms (microarray to RNA-seq). GREP^DR5^ predictions trained on cell lines, has been applied to pancreatic primary tumor xenograft (PTX) samples using microarray and RNA-seq data. *(A)* Performance of DR5+Casp8 predictor on 11 PTX models results in a PPV = 50%. *(B)* GREP^DR5^ prediction probability accurately predicts %T/C in 11 pancreatic PTX models (PPV = 100%). Additionally, GREP^DR5^ predictions correlates linearly with the anti-tumor activity (%T/C or %Regression) (Pearson’s R = -0.91, p = 10^−5^). *(C)* GREP^DR5^ predictions between the microarray and RNA-seq platforms are highly correlated (R = 0.87), showing that GREP can be readily used for translation to another platform. It should be noted that the data was not transformed (e.g. scaled or batch corrected) before applying the predictions.

The GREP^DR5^ model, which was derived from cell lines, was applied to expression values (Affymetrix, MAS5 normalized) from xenograft samples, without any additional transformations or scaling (S1 File). GREP^DR5^ correctly predicted all samples (PPV = 100%; AUC = 100%) (Fig 4B), in contrast to the DR5+Casp8 predictor, which had poor performance (PPV = 50%; AUC = 66%) (Fig 4A). Finally, even though GREP^DR5^ was trained using categorical sensitivity calls and excluding intermediate responders, the prediction probability correlated very well with the continuous activity measure (Pearson’s R = -0.91, p = 10^−5^).

We also present evidence that GREP^DR5^ predictions translate from Affymetrix microarray to RNAseq (Fig 4C). It should be noted again that we did not use any transformations or scaling of the RNAseq data before applying the GREP predictions. Standard translational approaches for gene expression involve transforming the data, using batch correction (mean shifts, z-scores, etc.) or batch correction [18], which requires a population of test samples, and cannot be performed on one or a few samples. This can be a severe limitation, especially for clinical biomarker assays. On the other hand, GREP’s expression ratios provide a significant practical advantage because they can be determined reliably even from one sample. These results show that ratios enable accurate and translatable predictions across model systems and even across platforms.

### Performance of GREP compared to standard approaches

**Fig 5:**
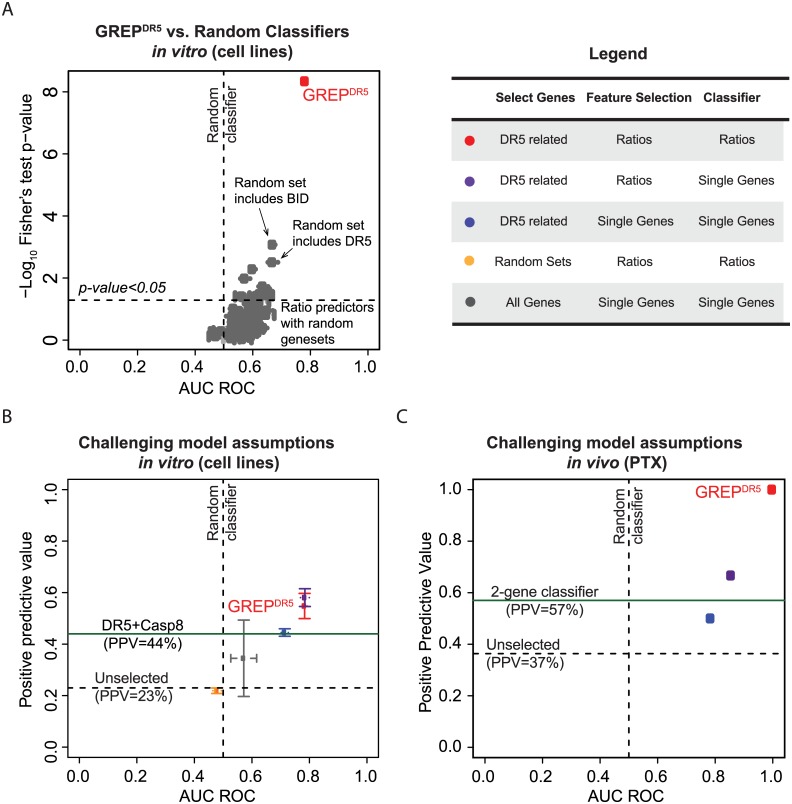
GREP improves predictions and translatability over standard approaches for feature selection and classification. (*A*) GREP^DR5^ compared to ratio classifiers with random gene sets (N = 100) of the same size as the hypothesis gene set. 90% of the models were not significantly better than random (Fisher’s test p-value>0.05). Examination of some random models that performed significantly showed that they included DR5-related genes. (*B*) Challenging the GREP modeling assumptions in predicting *in vitro* response. GREP^DR5^ was compared to four models, each built without one or more of its key assumptions (error bars show 95% confidence from cross-validation). GREP^DR5^ outperforms random and 2-gene classifiers, but a standard gene expression predictor that used ratios for feature selection performed just as well in cell lines. (*C*) Challenging the GREP modeling assumptions in predicting *in vivo* response. Validation of the classifier predictions in pancreatic patient-derived tumor xenograft models (PTX) compared to classifiers built with single genes. Assuming a 30% margin of error on the PPV calculation for 11 samples (95% confidence), GREP outperforms both the 2-gene classifier and a standard gene expression predictor that used ratios for feature selection. Vertical dotted line denotes AUC of random classifier (0.5).

Next, we explored how GREP performs compared to standard single gene classifiers. GREP outperforms standard approaches using single genes for feature selection and classification, and is not compromised by its core assumptions: hypothesis based gene selection; feature selection using ratios; and classification using ratios (Fig 5, S1 File). Both a standard gene expression classifier using all single genes as features (i.e., not hypothesis selected) and GREP^DR5^ based on random gene sets performed close to random (Fig 5A). A classifier built on hypothesis-based single genes performs like the DR5+CASP8 signature (PPV = 44%), while using ratios for feature selection, but only single genes for the classification step, yielded results which are comparable to GREP^DR5^ (PPV = 58%), as expected from a logistic regression approach (Fig 5B). Importantly, even though a standard gene expression classifier that used ratios for feature selection performed just as well for cell line predictions (Fig 5B), GREP^DR5^ was significantly superior to other models when translating to xenograft PTX models (Fig 5C) assuming a 30% margin of error on the PPV calculation for 11 samples (95% confidence). These results suggest that gene ratio classifiers are superior to classifiers built on absolute expression values, especially for translational sensitivity prediction.

### Interpretation of ratios in the GREP^DR5^ model

**Fig 6:**
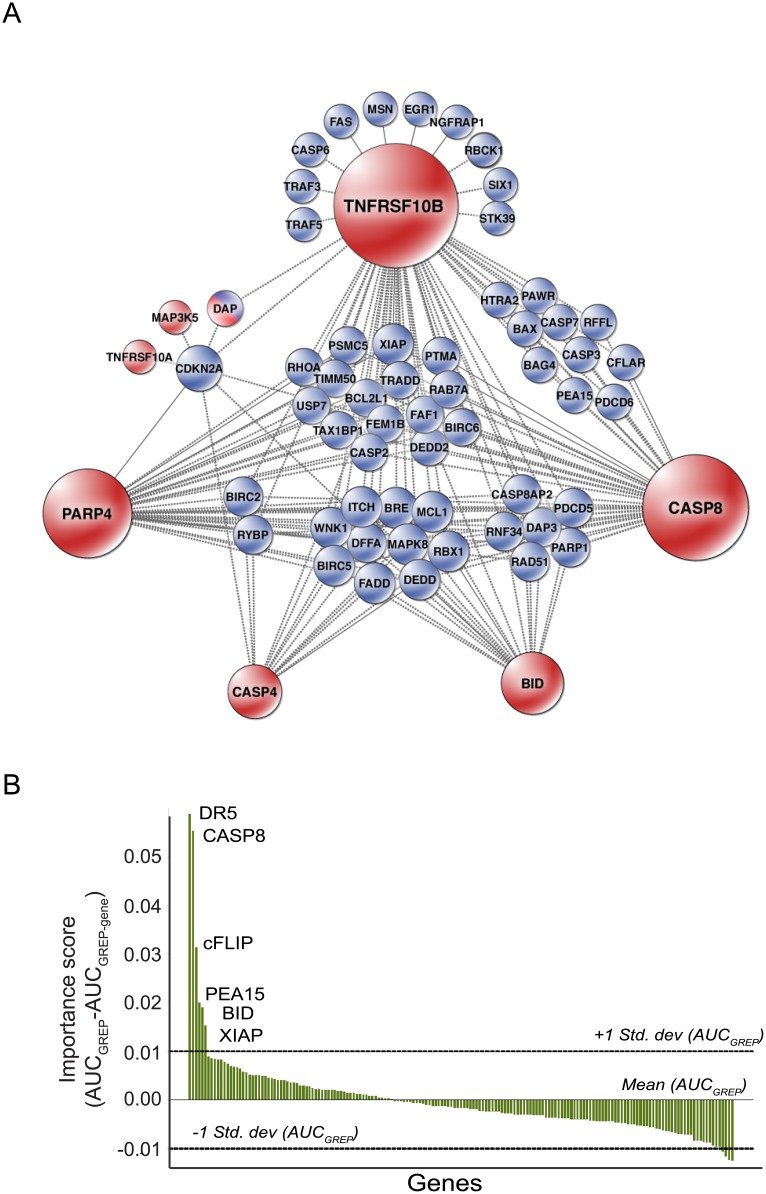
GREP reveals informative relationships between genes. (*A*) Network representation of ratios that significantly differentiate response identified by GREP^DR5^. Genes are connected if they are involved in a ratio, sized based on the number of ratios in which they appear, and colored based on their positivity (%times they appear in the numerator of ratios; ratios were ordered so that they are positively correlated with sensitivity). Red indicates positive, while blue indicates negative. Ratios used in the classifier are shown as bold connections. (*B*) Importance of individual genes in GREP^DR5^. Importance of individual features, each assessed using the receiver operator characteristics area under curve (AUC_ROC_) accuracy measure between the full GREP^DR5^ and one built with that feature excluded.

In order to facilitate interpretation of relevant ratios, we created a network visualization of all significant ratios in the GREP^DR5^ model by representing the component genes in a ratio as connected nodes (Fig 6A). Genes in a ratio are ordered so that the ratio is positively correlated with sensitivity. Nodes in the network sized based on the number of connections and are colored based on their positivity (proportion of times the gene is in the numerator). All the nodes, expect DAP1 are all either positive or negative. The positive nodes: TNFRSF10B, CASP8, PARP4, CASP4 and BID are also the largest nodes in the network. As expected, TNFRSF10B, CASP8, form key nodes in this network and are strongly positive regulators. BID, a substrate of CASP8 that mediates mitochondrial apoptosis in type II cells [5], also forms a key node as a proximal signaling mediator. CASP4, an inflammatory caspase [19], has previously been associated with Apo2L/TRAIL sensitivity in melanoma [20] and rheumatoid arthritis synovial fibroblasts [21], but otherwise is not widely associated with DR5 signaling and thus was an unexpected finding. Likewise, the role of PARP4, a vault poly (ADP) polymerase, in regulating the Apo2L/TRAIL pathway has not been previously demonstrated.

The majority of other genes are negative regulators, suggesting that most of this network is engaged in buffering apoptotic signaling, leading to multiple mechanisms of insensitivity to DR5 agonism. CDKN2A, a negative regulator connected to all five key nodes, is highlighted because it is exclusively connected to a few other genes. Its products, p16INK4a and p14ARF, are tumor suppressors implicated in the regulation of senescence and apoptosis through cell cycle and p53 pathways [22,23]. In particular, p16 has been shown to be epigenetically silenced in pancreatic cancer [24] highlighting the importance of measuring gene expression for predicting drug response.

To understand which genes may impact the performance of GREP^DR5^, we compared its cross-validation performance to 173 reduced GREP^DR5^ models, each with one gene omitted. TNFRSF10B and CASP8 were the two most essential genes for predictor performance, consistent with their critical role, as well as c-FLIP and XIAP, well established negative regulators of DR5 pathway signaling (Fig 6B). In addition to forming a key node, eliminating BID from the GREP^DR5^ list significantly (Standard deviation >1) reduced the prediction performance. This observation may suggest that activation of intrinsic apoptotic signaling is critical for response to DR5 agonism in cancer cells. Our elimination analysis of GREP^DR5^ genes also identified genes such as phosphoprotein-enriched-in-astrocytes 15 (PEA15), which has been previously associated with insensitivity to Apo2L/TRAIL in glioblastoma by modulation of the DISC to inhibit caspase-8 cleavage [25], but is otherwise not well described as a negative regulator of DR5. Thus, using ratios of genes to predict drug response can also be a powerful tool to uncover complex relationships between positive and negative regulators of target gene response.

## Conclusions

Despite compelling single agent anti-tumor activity in preclinical models of diverse tumor types, the reported clinical response to DR5 agonists in cancer patients has been restricted to a few partial responses in patients [3,4], highlighting a need to better predict DR5 agonist responsive patients. The development of a highly potent and selective DR5 agonist [10] enabled us to screen a large and diverse cancer cell line panel, facilitating the interrogation of the underlying molecular features required for response. Furthermore, in the pancreatic xenograft models, there was a striking linearity between prediction probability and anti-tumor response suggesting this method of response prediction is intimately tied to underlying tumor biology.

Although the genes selected for GREP^DR5^ had known relevance to DR5 or death domain signaling, the network visualization of ratios highlighted interactions important in the targeted pathway. Certain genes previously associated with TRAIL mediated sensitivity [15,26] such as GALNT14 or FUT3/6, did not associate with DR5 Nanobody response in our model. A possible explanation is that DR5Nb1-tetra may be less dependent on DR5 glycosylation for driving efficient receptor clustering as compared to Apo2L/TRAIL or antibodies. By contrast, all of the genes scoring in the GREP modelling are involved in apoptotic signaling.

Ratio classifiers offer the possibility of greater prediction accuracy [27,28] and improved translation to clinically applicable assays because they do not require establishing an expression threshold [16,29]. Nonetheless, ratio signatures may not be needed when single genes are strong predictors of drug response (e.g. vemurafenib response in BRAF^V600E^ melanoma) and thus may be most useful when the drug target, like DR5, is not an oncogenic driver or requires activation of forward signaling response. GREP methodology, specifically, allows for integration of multiple gene ratios into a robust classifier. GREP^DR5^ improved DR5Nb1-tetra response predictions over DR5 or CASP8 alone, especially for DR5-expressing insensitive lines, and identified important gene relationships in DR5 biology. In this respect, ratios may contain more information about the signaling state of the cell relative to absolute gene expression levels.

## Methods

### Automated Cell Line Screening

For the CLiP screen, DR5Nb1-tetra was maintained as a 6.7 μM stock solution. Prior to screening, it was serially diluted 2.5-fold in PBS with 0.001% bovine serum albumin and arrayed in single-use (1/day of screening) 1,536-well source plates (yielding a concentration range of 6.7 μM to 0.7 nM), sealed and frozen at -20°C. Cells were dispensed into 1,536-well assay plates (optimized for tissue culture) with a final volume of 5 μL and a concentration of 250 cells per well. 10 to 24 hours after plating, 15 nL of each dilution series was acoustically transferred to the 1,536-well assay plates using a Labcyte Echo 555, yielding final concentrations of 20 nM to 2.1 pM (11-point concentration-response assays). Assay plates were incubated at 37°C for 4 days. The remaining assay steps were the same as previously reported [12].

For the CRXX screen, DR5Nb1-tetra was transferred to CombinatoRx (now part of Horizon Discovery Inc.) as a PBS-diluted stock solution of 5 mg/mL and screening was performed as described [30]. Briefly, Nanobody was arrayed into 384-well source plates, sealed and frozen at -20°C until the day of screening. Cells were dispensed into 384-well assay plates with a final volume of 50 μL and cell concentrations (200–500 cells per well) individually optimized to detect growth in a 72 hour assay. Cell lines were tested in duplicate at three concentrations (1.0, 10, and 100 pM) of DR5Nb1-tetra. Diluted antibody was acoustically transferred in 2nL droplets from the source plate to the assay plates using a Labcyte Echo 555. After incubating at 37°C for 3 days, 100 ul/well of CellTiter-Glo reagent was added to the assay plates, and luminescence was recorded using an Envision plate reader.

### Xenograft Models

All animal studies were performed in accordance with the NIH Guide for the Care and Use of Laboratory Animals and the Novartis Institutes for Biomedical Research Animal Care and Use Committee guidelines. All studies were performed as previously described [10]. Briefly, human derived pancreatic tumor xenograft models (HPAX), surgical explants provided by NDRI or NCI following informed written consent, were passaged by subcutaneous implant of 10–15 mg tumor fragments (*in vivo* passage 4 to 6) from viably frozen or freshly explanted tumors. Tumor volumes (TV) were monitored twice weekly by calipering: TV(mm^3^) = [((l x w^2^) x 3.14159)) / 6]. When mean TV was ~100–200 mm^3^, mice were randomized to treatment groups as indicated. PBS or DR5Nb1-tetra were administered by tail vein injection (10 mL/kg) as indicated. Anti-tumor activity was reported as percent treatment/control (%T/C = 100 × ΔT_t_/ΔC_t_) or %Regression (%REG = 100 × ΔT_t_/T_i_ if ΔT_t_ < 0); where T = treated mean TV; C = control mean TV: i = initial; t = final; ΔT_t_ = T_i_—T_t_; and ΔC_t_ = C_i_—C_t_. Statistical analysis of anti-tumor activity was performed on ΔTV by ANOVA or Kruskal-Wallis, followed by post-hoc Tukey or Dunn’s test (Sigmaplot).

### Xenograft RNA Expression Profiling

RNA integrity was assessed with the Agilent 2100 Bioanalyzer (RNA 6000 Nano LabChip kit, Agilent Technologies). RNA samples with RIN score of > 7.0 were profiled. Microarray profiling was performed using the Human Genome U133 Plus2.0 gene chip array (Affymetrix). Probe synthesis, hybridization, washing, staining and scanning of the gene chips was performed according to the Affymetrix protocols. Probe level fluorescence intensities were normalized to an arithmetic mean (150) calculated with the Affymetrix Microarray Analysis Suite 5 (MAS5). Chip quality was accessed with selected quality-control parameters (background, % present calls, scaling factor and the 3’/5’ ratios of beta-actin and GAPDH reference genes) generated using the MAS 5.0 software.

### Xenograft RNA-seq Profiling

Methods used for RNA-seq profiling [31] were modified to align to both human reference GRCh37 as well as mouse reference genome and transcriptome mm10.

### Predictive modeling

MAS5 normalized (baseline 150) microarray data was used for the analysis. Probe-level data were converted to gene-level by using the best probe per gene [32]. Genes with low expression (75^th^% percentile <250) and dynamic range (Interquartile range <500) were filtered out. Ratios for all pairs of genes were computed for each sample. To limit ratio artifacts due to noise at low expression levels, a constant value was added to the numerator and denominator, after which the ratios were log-transformed. The association of log-ratios with sensitivity was examined using t-test statistic and significance (p-value) was determined using permutation tests (100 sample label permutations). False Discovery Rates (FDR) (q-values) were also estimated using permutation tests [33]. Ratios with FDR<0.1 were considered to be significant. Affinity propagation clustering was then used to identify clusters of the significant ratios and exemplars from each cluster [17]. Logistic regression was used as the classifier to identify prediction probabilities using a cutoff probability of 0.5 to define predicted sensitive and insensitive groups. Five-fold cross-validation was run on all the above steps to estimate the performance of GREP.

### Data analysis

Significance of association of categorical features (lineage, mutations), numerical features (expression, copy number) with sensitivity was assessed using a Fisher’s exact test, Student’s t-test, respectively. FDR calculations were performed using Benjamini-Hochberg method. All the analyses were performed using R 2.14.1. TIBCO Spotfire was used for visualization. Pathway enrichment was performed using Fisher’s exact test and significance cutoff was set at FDR<0.1 (MetaCore from GeneGo, Inc).

### Quantifying protein levels for DR5

Cells were collected with accutase (Innovative Cell Technologies, #AT104), washed twice with Assay Buffer: MACs rinse plus 0.05% BSA (Miltenyi, #130-091-222) and plated at 2x10^5 cells/well in a 96 well plate. Fc was blocked for 10 minutes in human blocking reagent (Miltenyi #120-000-442). Cells were pelleted and then resuspended in buffer containing either isotype (eBioscience #12-471-42) or DR5 (eBiosciences #12-9908-73) PE conjugated antibodies. Incubation occurred for 30 minutes on ice. Cells were then washed twice in assay buffer, resuspended in assay buffer containing 7AAD at 10ul/ml (eBiosciences #00-6993-50) and analyzed via FACS on a Canto (BD Biosciences). Single, live, 7AAD cells were gated. The resulting PE MFI was determined with isotype subtracted from DR5 signal. All cell lines could not be run at one time; therefore Colo205 was included in each run to determine consistency between runs and to be used as a reference point. All cell lines were analyzed two to three times. The MFI for each cell line was then categorized based on the Colo205 control. Cell lines with MFI within 30% of Colo205 were considered similar in expression (medium level). Greater than 30% were considered higher expression (high level), and less than 30% were considered lower expression (low level).

## Supporting Information

S1 Fig Representation of hematopoietic and lung lineages assed in *in vitro* screens. Pie-chart showing composition of tumor subtypes in hematopoietic and lymphoid and lung lineages in our *in vitro* screen tested for response to DR5Nb1-tetra.
(EPS)

S2 Fig Representation drug activity curve. A_max_ is defined as the maximum % inhibition of ATP metabolism measured in treated cells compared to untreated controls, where 0% corresponds to growth at the same rate as in untreated wells, 100% corresponds to total kill, and 50% corresponds to the treated wells containing only half the population seen in the untreated wells (in our case assessed using metabolic activity). EC_50_ is the sigmoidal model’s inflection point concentration, occurring at half the A_max_ inhibition level, and IC_50_ is the concentration where the sigmoidal fit crosses 50% inhibition (set to the maximum tested when A_max_ corresponded to less than 50% inhibition).
(EPS)

S3 Fig Differential association of copy number and mutations with sensitivity to DR5NB1-tetra. Copy number and mutation features correlated with sensitivity. (*A*) Differential analysis of copy number features shows that high CN of the 8p21.3 (chromosomal region containing DR5, DR4, etc.) is significantly associated with sensitivity. (*B*) Differential analysis of mutation features did not yield any significant results (FDR<0.1). However, several features like CDKN2A, MET, KRAS, TP53 are nominally significant (p<0.05).
(EPS)

S4 Fig Simulation experiments showing the rank distribution of DR5 and Casp8 expression by down sampling the number of cell lines tested for sensitivity to DR5Nb1-tetra. DR5 and caspase-8 would most likely not have been identified by screening fewer numbers of cell lines. Results of a simulation experiment performed by resampling fewer (100 or 200) samples from the *in vitro* screens and running differential analysis using all genes, shows that (*A*) DR5 and (*B*) Casp8 are ranked on average >1000 and are nominally significant (p-value<0.05) in ≤25% or ≤10% of the runs, respectively.
(EPS)

S5 Fig GREP^DR5^ prediction probability in cell lines across lineages. GREP^DR5^ performs well across lineages irrespective of the response rate of individual lineages. GREP^DR5^ prediction probabilities are plotted for lineages (sorted by response rate). GREP^DR5^ model which was built across all lineages performs well (high positive predictive value) across a majority of the lineages on either end of the response rate spectrum. However, GREP^DR5^ has lower sensitivity (number of predicted sensitives/number of sensitives) in some lineages like glioma, lung and breast.
(EPS)

S6 Fig Anti-tumor tumor activity of DR5nb1-tera against pancreatic PTX models. DR5Nb1-tetra response (red) in 11 primary pancreatic tumor xenografts compared to vehicle (black) shows activity (stasis or regression) in 37% (4 out of 11) models.
(EPS)

S7 Fig Significant ratios in the GREP^DR5^ model. PDF file showing scatter plots of all pairs of genes appearing in a ratio in the GREP^DR5^ model.
(PDF)

S1 Table Pathway analysis of significant differential genes. Pathway analysis of significant differential genes (FDR<0.05) revealed enrichment of the apoptosis pathway (MetaCore from GeneGo, Inc.).
(XLSX)

S2 Table Hypothesis gene set used for GREP modeling. List of 173 hypothesis genes used for building the Gene Expression Ratio Prediction for DR5, GREP^DR5^. These features were selected based on their reported relevance to DR5 signaling, sensitivity to a DR5 agonist, or involved in death domain caspase signaling (MetaCore from GeneGo, Inc.).
(XLSX)

S1 File Supplementary data tables: **In-vitro results**. Excel table showing for each line the IC_50_, Amax, experimental sensitivity call, MAS5 gene expression value, 2-gene prediction, and GREP^DR5^ prediction for all genes used in the GREP model. **All genes**. Excel table showing differential analysis of all genes with DR5Nb1-tetra sensitivity calls. **GREP model classifier**. Excel table showing coefficients for ratios used in the GREP^DR5^ model. **In-vivo data**. Excel table showing for each primary tumor xenograft model, the T/C, sensitivity call, GREP prediction and gene expression for all genes used in the GREP model.
(XLSX)
