# Correction: Gene Expression Ratios Lead to Accurate and Translatable Predictors of DR5 Agonism across Multiple Tumor Lineages

**DOI:** 10.1371/journal.pone.0146635

**Published:** 2016-01-05

**Authors:** Anupama Reddy, Joseph D. Growney, Nick S. Wilson, Caroline M. Emery, Jennifer A. Johnson, Rebecca Ward, Kelli A. Monaco, Joshua Korn, John E. Monahan, Mark D. Stump, Felipa A. Mapa, Christopher J. Wilson, Janine Steiger, Jebediah Ledell, Richard J. Rickles, Vic E. Myer, Seth A. Ettenberg, Robert Schlegel, William R. Sellers, Heather A. Huet, Joseph Lehár

S1 File includes the incorrect data in the “Data S3 GREP model classifier” tab and in Column F (GREP prediction) of the “Data S4 in-vivo data” tab, which are from another model not discussed in this paper.

Please see the corrected S1 File here. The authors confirm that all results and figures in the original work remain unaffected by this error and correspond to the updated supplementary data in this correction.

## Supporting Information

S1 File Supplementary data tables: In-vitro results. Excel table showing for each line the IC_50_, Amax, experimental sensitivity call, MAS5 gene expression value, 2-gene prediction, and GREP^DR5^ prediction for all genes used in the GREP model. **All genes**. Excel table showing differential analysis of all genes with DR5Nb1-tetra sensitivity calls. **GREP model classifier**. Excel table showing coefficients for ratios used in the GREP^DR5^ model. **In-vivo data**. Excel table showing for each primary tumor xenograft model, the T/C, sensitivity call, GREP prediction and gene expression for all genes used in the GREP model.
(XLSX)
