# Supplementary material for: Gene Expression Ratios Lead to Accurate and Translatable Predictors of DR5 Agonism across Multiple Tumor Lineages
Source: PLoS One. 2015 Sep 17;10(9):e0138486. doi: 10.1371/journal.pone.0138486 (PMC4574744; doi:10.1371/journal.pone.0138486)

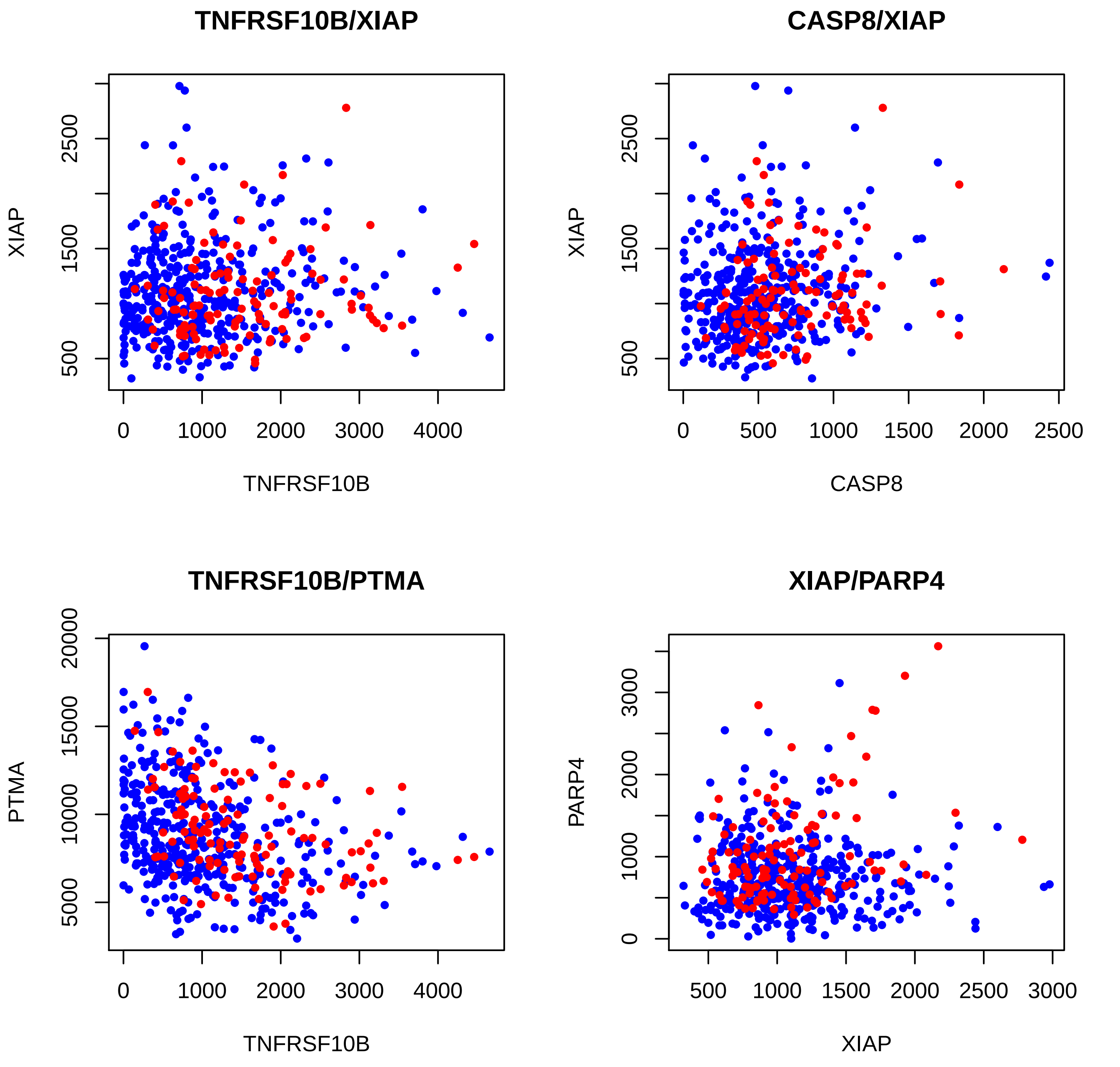

TNFRSF10B/XIAP
XIAP
TNFRSF10B
500
1500
2500
0
1000
2000
3000
4000
CASP8/XIAP
XIAP
CASP8
500
1500
2500
0
500
1000
1500
2000
2500
TNFRSF10B/PTMA
PTMA
TNFRSF10B
5000
10000
15000
20000
0
1000
2000
3000
4000
XIAP/PARP4
PARP4
XIAP
0
1000
2000
3000
500
1000
1500
2000
2500
3000

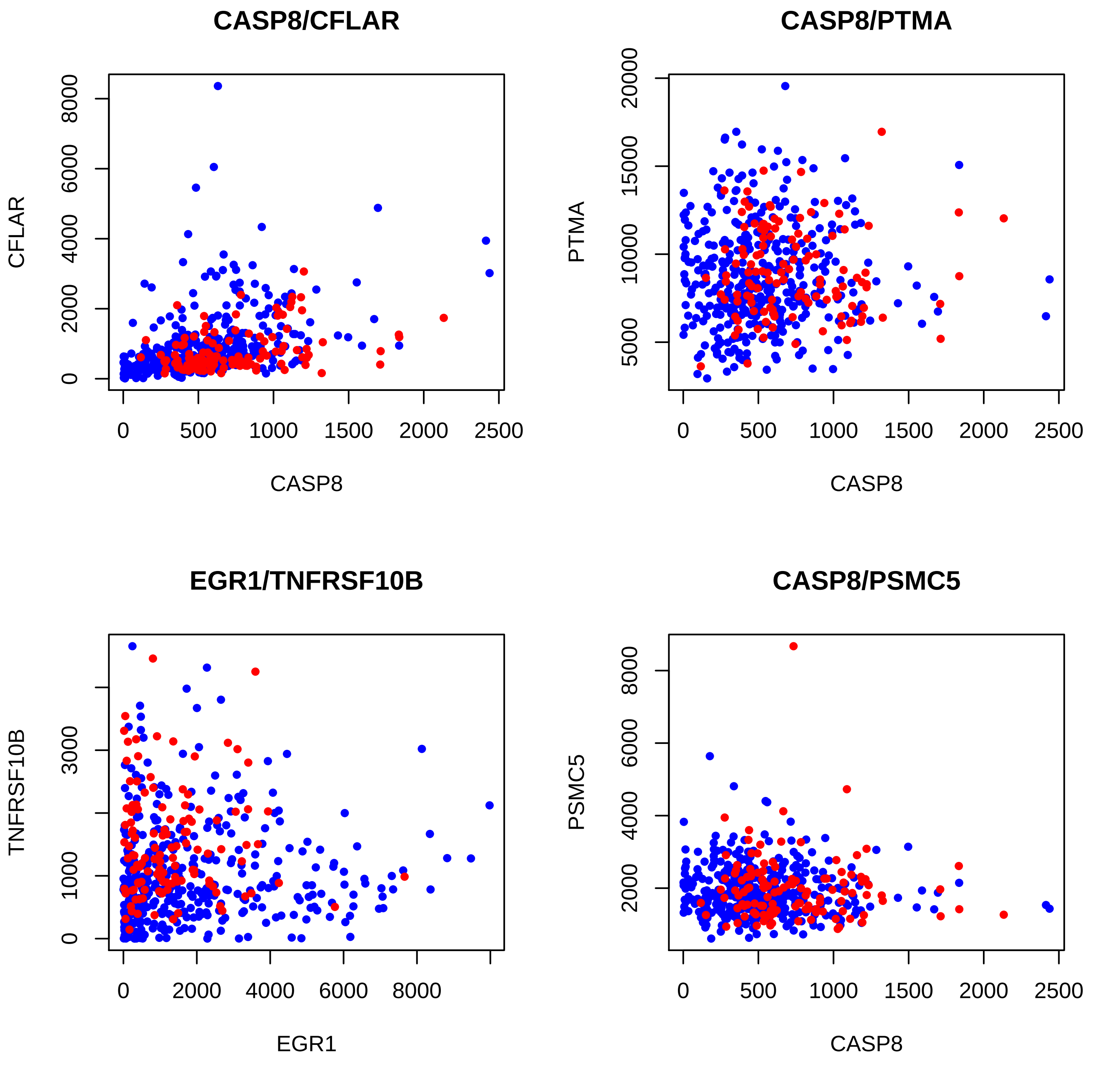

CASP8/CFLAR
CFLAR
CASP8
0
2000
4000
6000
8000
500
1000
1500
2000
2500
CASP8/PTMA
PTMA
5000
10000
15000
20000
EGR1/TNFRSF10B
TNFRSF10B
EGR1
1000
3000
8000
CASP8/PSMC5
PSMC5

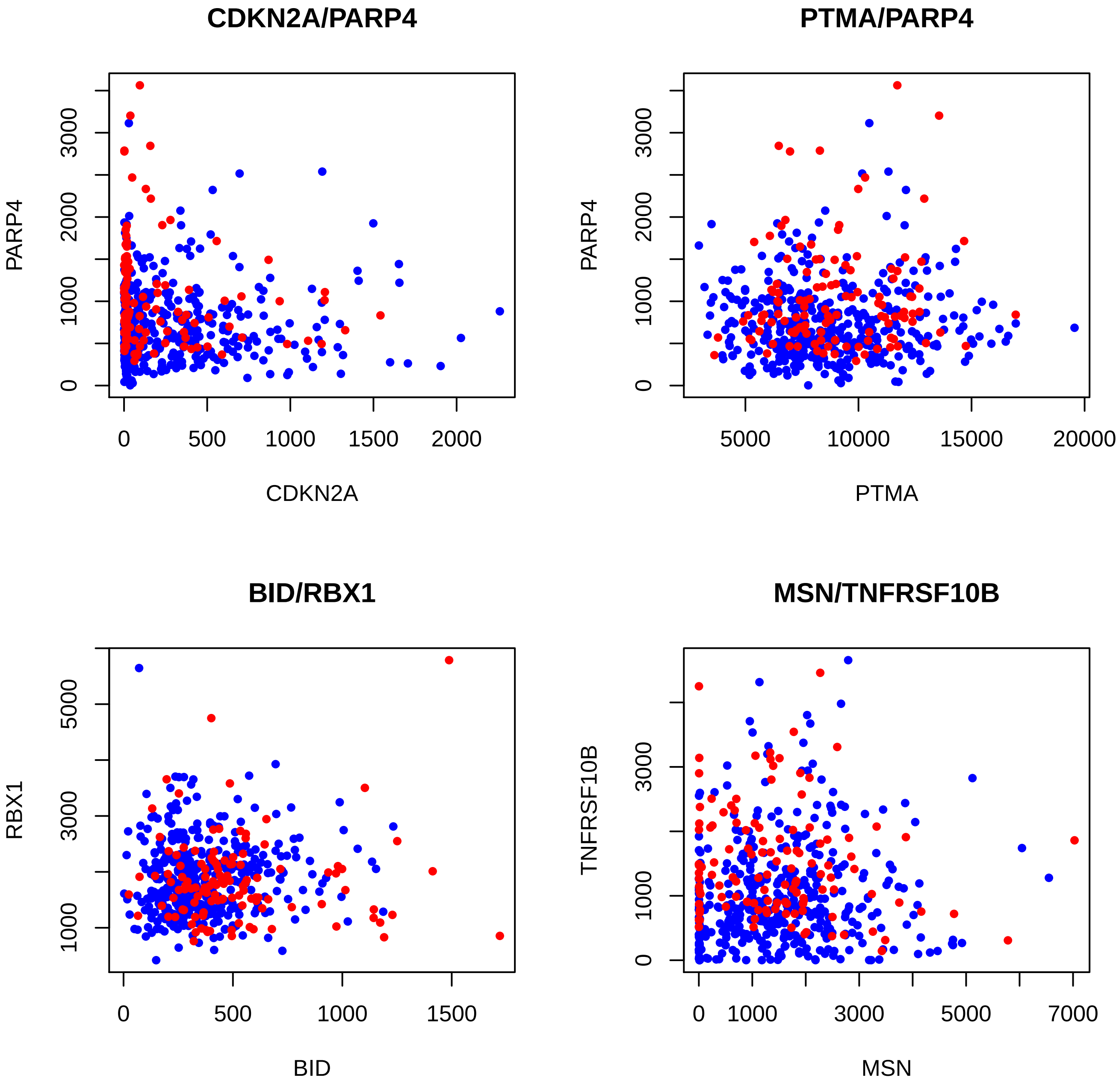
CDKN2A/PARP4
PARP4
CDKN2A
0 500 1000 1500 2000
0 1000 2000 3000
PTMA/PARP4
PARP4
PTMA
5000 10000 15000 20000
0 1000 2000 3000
BID/RBX1
RBX1
BID
0 500 1000 1500
1000 3000 5000
MSN/TNFRSF10B
TNFRSF10B
MSN
0 1000 3000 5000 7000
0 1000 3000

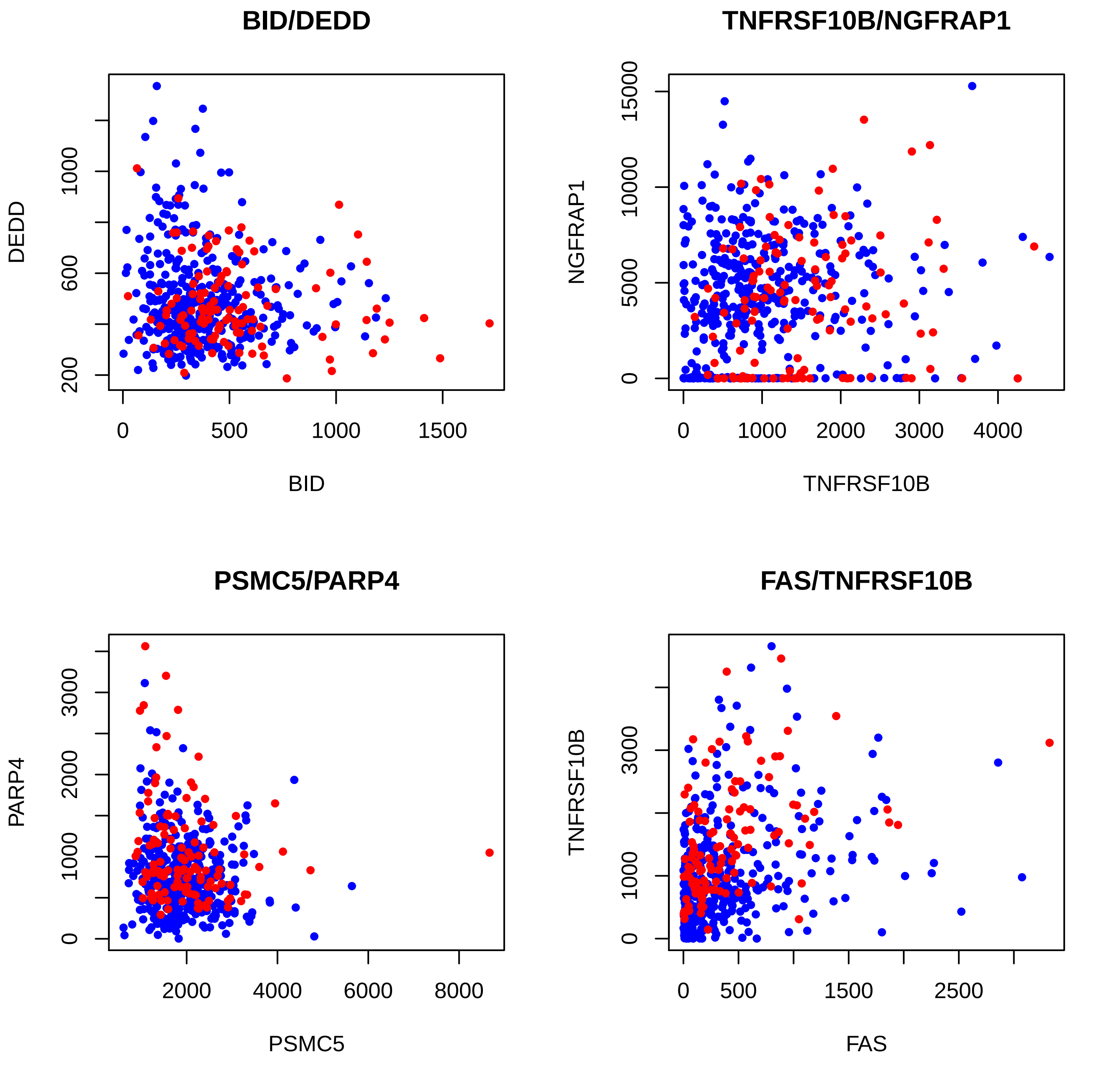

BID/DEDD
DEDD
BID
200
600
1000
0
500
1000
1500
TNFRSF10B/NGFRAP1
NGFRAP1
TNFRSF10B
0
5000
10000
15000
0
1000
2000
3000
4000
PSMC5/PARP4
PARP4
PSMC5
0
1000
2000
3000
2000
4000
6000
8000
FAS/TNFRSF10B
TNFRSF10B
FAS
0
1000
3000
0
500
1500
2500

CASP4/DEDD

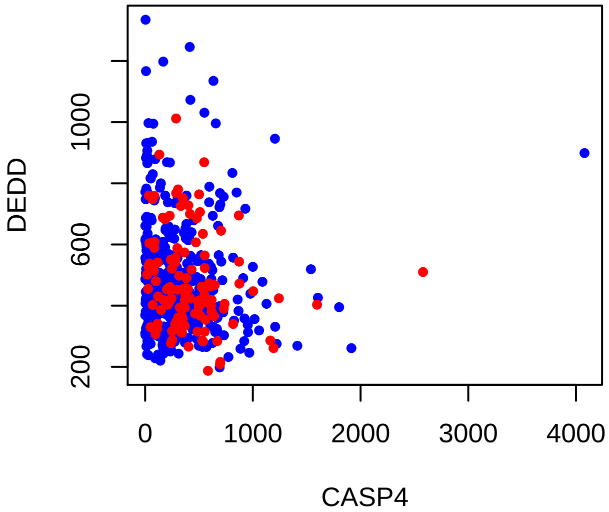

Supplement: S7 Fig — PDF file showing scatter plots of all pairs of genes appearing in a ratio in the GREPDR5 model. (PDF) [file pone.0138486.s007.pdf]
